# COVID-19 pandemic: The African paradox

**DOI:** 10.7189/jogh.10.020348

**Published:** 2020-12

**Authors:** Debajyoti Ghosh, Jonathan A Bernstein, Tesfaye B Mersha

**Affiliations:** 1 Division of Immunology, Allergy & Rheumatology, Department of Internal Medicine, Cincinnati Children’s Hospital Medical Center, University of Cincinnati, Cincinnati, Ohio, USA; 2 Division of Asthma Research, Department of Pediatrics, Cincinnati Children’s Hospital Medical Center, University of Cincinnati, Cincinnati, Ohio, USA

Since COVID-19 became a pandemic, projection models were developed for Africa, with the assumption that SARS-CoV-2 has an exponential pattern of transmission. Crowded social life and poor personal hygiene in Africa can be conducive for COVID-19 spread. However, as of July 20, 2020, only about 9691 COVID-19 deaths have been reported from African continent among a population of 1.34 billion (compared to 143 000/328.2 million in the US alone). Although the number of infected subjects increased considerably during mid-July 2020 reaching about 597223 confirmed cases, the case fatality remained remarkably low in Africa [1]. According to the WHO, COVID-19 in Africa will likely “smoulder” ie, spread from hotspots at a slow, but steady pace rather than exponentially as elsewhere worldwide (https://www.afro.who.int/news/new-who-estimates-190-000-people-could-die-COVID-19-africa-if-not-controlled; last accessed July 21, 2020). Whether this paradox is due to genetics and immunity, comparatively young population, lower rates of comorbidities or just due to limited testing and late arriver of the pathogen in the continent, the low COVID-19 morbidity in African countries, with its fragile health care system, continues to puzzle experts. Herein, we outlined current concepts about potential players behind this low COVID-19 related mortality in Africa.

## TRACKING SARS-COV-2 IN AFRICA

Since SARS-CoV-2 was introduced at different time points in different countries, an approach to capture the COVID-19 trajectory would be tracking the number of infected cases from the day of the first reported case in countries of multiple continents. This approach, although confounded by multiple pathogen- and host-specific factors, could be useful for obtaining comparative insights into COVID-19 infection across countries. A major pathogen-specific factor associated with SARS-CoV-2 spread in different continents is the types of circulating strains/mutants. Whereas numerous SARS-CoV-2 genome sequences have been submitted from the West (about 13 308 sequences from the US, UK 32 604, Spain 1847), only about 356 have been reported from South Africa and none from several other African countries. Another challenge in capturing the COVID-19 landscape in Africa is the lower number of tests (per thousand people) performed in African countries (South Africa 41.68, Zimbabwe 2.79, Kenya 4.54, Ethiopia 2.18) compared to European (UK 118.58, Italy 103.57, Spain 87.13) and North American (USA 138.17, Canada 93.28) countries. Expanding testing capability across Africa is therefore warranted.

## CLIMATE HYPOTHESIS

**Figure 1:**
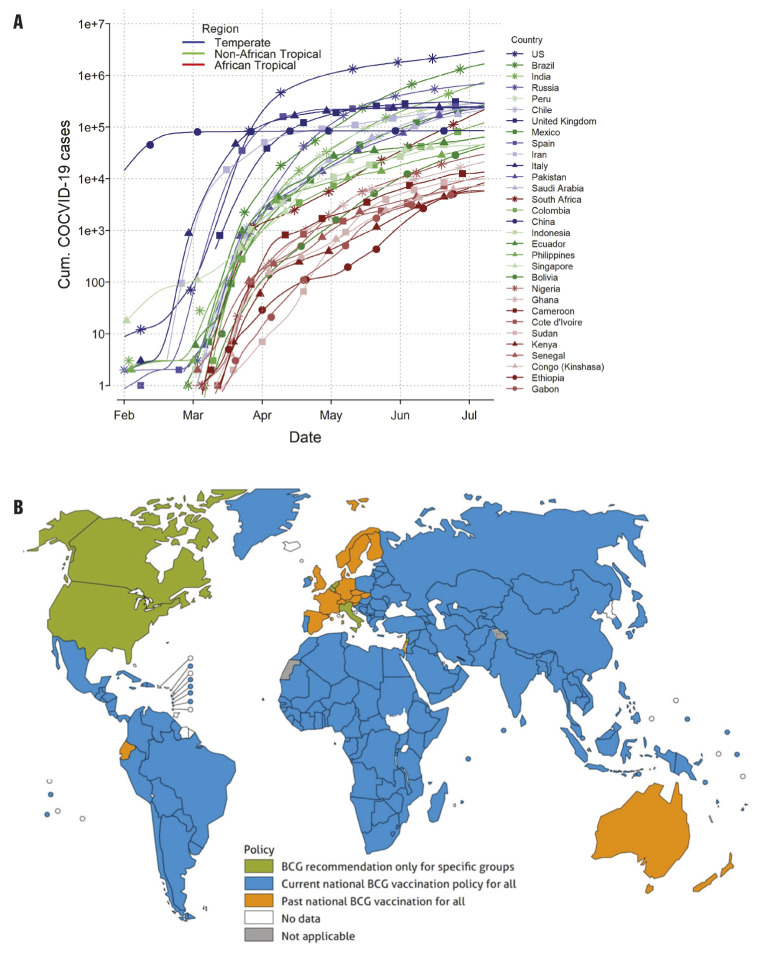
COVID-19 prevalence: Geographic variations and vaccination coverage. **Panel A**. Spread of COVID-19 in Africa vs countries in the temperate region. There is significant different in cases including trajectories between the two regions. Numbers of total cases have been plotted against days since first reported case in respective countries (as of 5/15/2020). The spread of COVID-19 in Africa vs the rest of the tropical countries. The graph depicts different trajectories due to environmental variation. Numbers of total cases have been plotted against days since first reported case in respective countries (as of 7/07/2020). The COVID-19 pandemic has shown a markedly low proportion of cases among young people, and this could be the reason why COVID-19 infection rate is the lowest in African countries except South Africa. COVID-19 infections are rising sharply in South Africa. South Africa resembles the countries of South America more than those of its home continent. **Panel B**: Bacillus Calmette-Guérin (BCG) vaccination coverage map by country. Data from the World Health Organization about the BCG coverage in each country showing global status of the BCG vaccination program [3]. In contrast to the countries with no active BCG vaccination program (including the current COVID-19 epicenters such as the US, Italy, Spain, Ecuador), countries with active BCG vaccination program of African countries could be effective in the fight against COVID-19.

It has been argued since most human coronavirus infections associated with common cold symptoms peak in the winter months (December – April), and are undetectable during summer months in temperate regions of the world, SARS-CoV-2 infection will diminish as temperatures rise in the summer. Indeed, a recent study used a weather model to predict regions associated with a higher risk of COVID-19 community spread [2]. The high-risk temperate Western country zones and South Africa which have 5-11°C mean temperatures and 47%-79% relative humidity have more COVID-19 cases than tropical African countries (**Figure 1**, Panel A). In contrast, tropical Asian and Latin American countries are disproportionately infected by SARS-CoV-2 compared to tropical African countries (**Figure 1**, Panel A) suggesting other determinants than climate alone impact the spread of SARS-CoV-2. For example, living in high altitudes (less prone to hypoxia) appears to reduce transmission and death rates from COVID-19. So far, COVID-19 pandemic has shown a markedly low proportion of cases among young people, and this could be the reason why COVID-19 death rate is the lowest in African countries except South Africa. COVID-19 infections are rising sharply in South Africa which resembles the countries of South America more than those of its home continent (**Figure 1**, Panel A).

## THE GENETIC HYPOTHESIS

Among host factors, the ACE-2 gene which encodes angiotensin-converting enzyme-2 (ACE-2), the receptor for SARS-CoV2, remains a key determinant. Since individuals with African ancestry don’t respond as well to ACE inhibitors compared to calcium blockers and β-adrenergic blocker anti-hypertensives [4], one can hypothesize that this decreased response could be potentially linked to low COVID-19 prevalence in Africa. Racial differences between black and white groups in ACE-2 gene polymorphisms, might differentially affect ACE-2 receptor activity and COVID-19 treatment response. The human serine protease gene TMPRSS2, which primes the SARS-CoV-2 spike protein and facilitates its binding to host cells, represents another critical host factor regulating infection.

## EVOLUTION BY NATURAL SELECTION HYPOTHESIS

Ancestors of modern humans lived and co-evolved with viruses and other pathogens in environments where infectious tropical diseases induced a strong immuno-genetic selection. Early in human evolution, parasite expulsion in the African tropics, including malaria, was a dominant immunogenetic selective force in shaping human survival. For example, the Fulani people of West Africa have fewer malaria cases, lower prevalence of *Plasmodium falciparum* infection and altered Th1/Th2 cytokine responses compared to their geographic neighbors. Sickle cell anemia (caused by hemoglobin-Beta gene mutations) and East African sleeping sickness (linked to the apolipoprotein L1 genetic variant; APOL1) gene represent two examples where environmental conditions have selected some alleles for local adaptation as protection from indigenous diseases like malaria and Trypanosoma parasitism, respectively. However, this adaptation is not without unintended consequences as sickle cell disease leads to painful crisis leading to organ damage whereas the APO1 variant is associate with increased susceptibility to chronic kidney disease (CKD). These evolutionary protective changes are supported by findings that the APO1 variant is missing among inhabitants of the Ethiopian highlands where the Tsetse fly vector for the trypanosome parasite is absent paralleling a very low incidence of CKD. Thus, prolonged co-occurrence and exposure of Africans to fatal pathogens elevates allelic frequencies that confer protection against infectious diseases. Altered disease-relevant allele frequency resulting from strong selection pressure, altered Th1/Th2 balance and kidney function could play critical roles in determining the COVID-19 trajectory in Africa.

**Figure:**
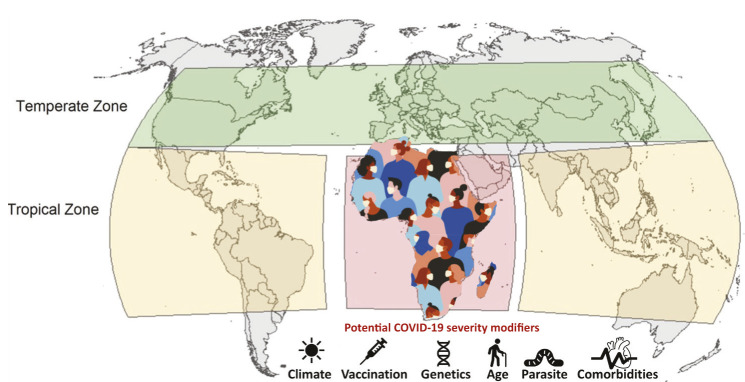
Photo: COVID-19 in Africa (from the author’s own collection, used with permission).

## THE TRAINED IMMUNITY HYPOTHESIS

The hypothesis of ‘trained immunity’ is defined as “memory-like properties of innate immune cells predominantly originating from exposure to a primary stimulus like a vaccine”. An example is the Bacillus Calmette-Guérin (BCG) vaccination, primarily administered against tuberculosis, which could play a protective role by promoting herd immunity against SARS-Cov-2. It has been argued that countries currently or previously using BCG vaccinations to prevent tuberculosis in neonates might have secondary protective benefits against COVID-19. Interestingly, African countries with an ongoing BCG vaccination policy have low COVID-19 spread in contrast to North American and EU countries which have ended BCG vaccination and are now epicenters of the COVID-19 pandemic (**Figure 1**, Panel B) [5]. The mechanism of this “pathogen-agnostic” effect conferred by live attenuated vaccines (eg, BCG, measles), although incompletely understood, may include cross-reactive T cells (eg, heterologous immunity) and innate immune components (eg, trained immunity) in neonates and older infants [6].

Multiple clinical trials listed in clinicaltrials.gov have linked BCG vaccination with COVID-19. Previous studies involving randomized low-weight infants in Guinea-Bissau (West Africa) indicated a significant reduction in mortality rate potentially due to enhanced immune defense against septicemia and pneumonia [7,8]. Castro et al retrospectively analyzed 464 611 hospitalization episodes (1992-2011; 25 years) and found hospital admissions due to respiratory infections unrelated to tuberculosis was significantly lower in BCG vaccinated vs non-BCG vaccinated children of all age groups (total preventive fraction = 41.4%; 95% CI = 40.3-42.5; *P* < 0.001) [9]. Arts et al demonstrated that BCG induces genome-wide epigenetic reprogramming of human monocytes (trained immunity) that protected against experimental viral infection predominantly via the IL-1 pathway [10]. SARS-CoV-2 infection can increase ‘acute respiratory distress syndrome’ (ARDS) associated with ‘cytokine storm’ which is characterized by dysregulated overexpression of proinflammatory cytokines, particularly IL-1β, IL-6 and TNFα, leading to increased vascular permeability, coagulopathy, organ damage and multiorgan failure. Since BCG vaccination induces epigenetic changes in immune cells, followed by functional reprogramming resulting in altered cytokine responses (particularly IL-1β), it might confer a protective effect against SARS-CoV-2. Therefore, identifying the genetic variants responsible for the modulation of immune responses is necessary to obtain further insights into associated immune reactions and clinical predisposition to SARS-CoV-2 infection. In addition to trained immunity, parasitic infections such as schistosomiasis confer potential beneficial effects against COVID-19 (*P* < 0.001) [11].

## THE AGE FACTOR HYPOTHESIS

Africa has the largest concentration of young people in the world. According to the United Nations, 226 million youth aged 15-24 lived in Africa in 2015 representing nearly 20% of Africa’s population. If one includes all people aged below 35, this number increases to a staggering three quarters of Africa’s population. Interestingly, human immunity against pathogens is highly influenced by age, particularly via immunosenescence, a mechanism defined as the “progressive deterioration of the immune system with aging” [12] This process dampens the generation of protective B and T cell-mediated adaptive immunity resulting in increased disease susceptibility and severity in the elderly population. Older adults may also exhibit the ‘senescence associated secretory phenotype” of non-lymphoid cells (associated with high baseline serum concentrations of C reactive protein, interleukin-6, interleukin-8 and chemokines) making them unable to properly clear dead and dying cells resulting in accumulation of senescent cells in organs causing organ damage [13].

The frequency of aged populations (age ≥60 years per thousand individuals) is remarkably lower in case of African countries (frequencies 3.385, 2.686 and 3.528 for Ghana, Kenya and Ethiopia respectively) compared to western countries (23.021, 21.228 and 18.517 for Italy, France and USA, respectively). Therefore, Africa’s predominantly younger and rural population with resultant fewer high-risk comorbidities may modify the severity of the COVID-19 epidemic. The large youth population may lead to more infections but most of these infections will be asymptomatic or mild and probably will go undetected.

## CONCLUSION

Multiple factors such as climate, genetic background, parasite load, vaccination status and stage of the pandemic have confounded our ability to elucidate the potential protective role of vaccination-induced trained immunity against COVID-19 in African countries. Differences in demographics (low median population age in Africa, compared to the West), sex, comorbid disease burden, testing rates, test bias, diagnostic issues and genetic sequences of circulating SARS-CoV-2 strains should be also considered [14].

Collectively, although Africa has suffered fewer COVID-19 cases until now, African governments and health care workers should not become complacent as future waves may be increasingly difficult to contain due to lack of preparedness. Notably, the 1918 flu pandemic killed approximately 2.4 million Africans, ie, 1.8% of the continent’s population (https://encyclopedia.1914-1918-online.net/article/influenza_pandemic_africa). According to the UN, COVID-19 has spread to all African countries with projections of 3.6–5.5 million hospitalizations, including 52-107 000 cases requiring active breathing support (https://www.un.org/africarenewal/news/coronavirus/new-who-estimates-190-000-people-could-die-COVID-19-africa-if-not-controlled; last accessed July 19, 2020). However, across all 54 countries, on average, 3.10 ICU beds and 0.97 ventilators per 100 000 people are available for use [15]. Furthermore, the slowdown of the world economy due to COVID19 will have a direct catastrophic effect on Africa’s economies and health capacity. The new “norm” should be preventive interventions such as contact tracing, social distancing, hand washing, surface sanitization and universal mask use (especially when distancing is not possible) until coronavirus vaccinations become available. In addition, collecting viral genome sequences along with relevant population data should be encouraged to effectively combat the devastating health and economic consequences related to COVID-19.
